# Supplementary material for: Anti-Idiotype scFv Localizes an Autoepitope in the Globular Domain of C1q
Source: Int J Mol Sci. 2021 Aug 1;22(15):8288. doi: 10.3390/ijms22158288 (PMC8347764; doi:10.3390/ijms22158288)
Supplement: Supplementary file 1 [file ijms-22-08288-s001.zip › ijms-1308440-supplementary.pdf]

Supplementary Material

Table S1. Data representing the plot in Figure 1 for each of the partially purified anti-idiotypic scFv clones analysed as inhibitors of the recognition of immobilised C1q by IgG_{LN}.with SD values.

scFv clone	0 µg		2.5 µg		5 µg		10 µg	
	% binding	SD	% binding	SD	% binding	SD	% binding	SD
**A1**	**100**	**0**	**57**	**1.6**	**52**	**1.4**	**53**	**2.4**
A3	100	0	117	13.5	86	25.3	70	12.5
A5	100	0	101	0	103	28	112	18
A8	100	0	99	0	91	6	101	9
A9	100	0	110	4.3	95	6.5	72	19.2
A12	100	0	85	1.2	67	2.3	67	21.2
B2	100	0	97	0	92	10	88	3
B6	100	0	91	0	83	9	81	7
C5	100	0	97	0	91	5	97	6
C9	100	0	100	0	101	7	86	5
D8	100	0	105	0	123	9	101	3
E1	100	0	102	0	102	5	115	10
F6	100	0	72	0	56	14	44	3
F9	100	0	68	0	48	7	44	5
